# How Does a Cyclops Lesion Impact Anterior Cruciate Ligament (ACL) Reconstruction Recovery?

**DOI:** 10.7759/cureus.80138

**Published:** 2025-03-06

**Authors:** Victoria M Estevez, Paul Danahy, Jonathan Whittington, Michael Ferrell

**Affiliations:** 1 Orthopaedic Surgery, Lake Erie College of Osteopathic Medicine Bradenton, Bradenton, USA; 2 Orthopaedic Surgery, Ortho Sport and Spine Physicians, Atlanta, USA

**Keywords:** acl injury, anterior cruciate ligament (acl) reconstruction, cyclops lesion, flexion contracture, manipulation under anesthesia

## Abstract

This case explores the complexities of treating a flexion restriction in a patient following anterior cruciate ligament (ACL) reconstruction, medial meniscus repair, and lateral meniscectomy. The aim is to provide insights into the treatment pathway when a flexion restriction develops post-ACL reconstruction and fails to improve with nonoperative measures. The patient, a 16-year-old high school track athlete, sustained a torn right ACL in a car accident and presented with severe knee instability, difficulty climbing stairs, and pain during ambulation. This case highlights the use of various nonoperative treatment strategies to address a flexion restriction that began developing within one month postoperatively. Despite aggressive physical therapy, the use of a knee extension machine, a brace locked in extension, and steroid treatment, the patient exhibited a persistent flexion restriction of five degrees, eventually necessitating surgical intervention. Ultimately, the patient achieved full recovery after undergoing arthroscopic lysis of adhesions and manipulation under anesthesia performed 10 months after the initial ACL reconstruction.

## Introduction

The anterior cruciate ligament (ACL) is one of the four stabilizing ligaments in the knee and functions to prevent anterior translation of the tibia relative to the femur [1]. In addition to its role in knee stabilization, the ACL also restricts excessive rotational movements to protect the surrounding menisci [2]. Athletes commonly experience ACL tears due to direct trauma or, at times, overuse, often requiring medical intervention to return to their sport. However, ACL injuries are not confined to the sports arena; they can also occur during motor vehicle accidents (MVAs), where abrupt force and impact may hyperextend the knee beyond its natural range of motion [3]. In such accidents, the knee is subjected to unnatural stresses similar to those experienced during athletic maneuvers, leading to ligament rupture. Whether occurring on the field or in a collision, ACL injuries result from excessive torque, sudden deceleration, and compromised joint stability, which explains their prevalence in both athletes and trauma victims.

As with any surgery, complications can arise following ACL reconstruction. In this case, the patient developed a cyclops lesion, a fibrous nodule composed of granulation tissue, fibrocartilage, and occasionally bony fragments [4]. The formation of cyclops lesions is triggered by the inflammatory process associated with the body's natural healing. However, in this case, excessive tissue growth led to a lesion that caused extension loss due to mechanical obstruction [4]. This case describes a complete ACL tear, repaired through reconstruction, which was complicated by a cyclops lesion in a female high school track athlete following an MVA.

## Case presentation

**Figure 1:**
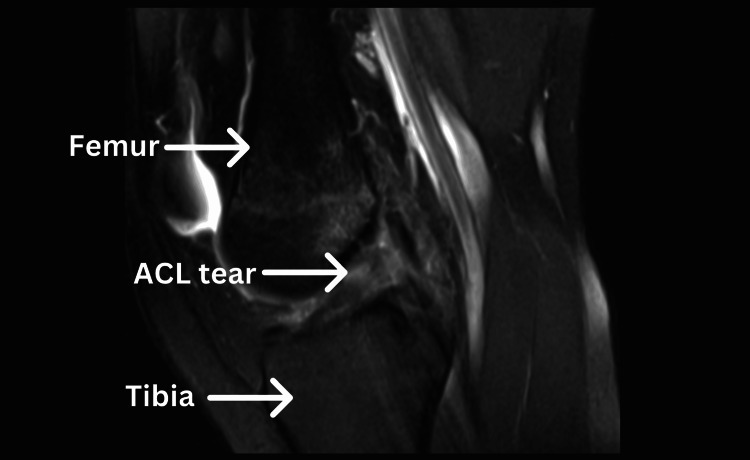
Magnetic resonance imaging (MRI) showing the patient’s torn ACL, along with the labeled femur and tibia ACL: Anterior cruciate ligament.

A 16-year-4-month-old African American female track athlete presented with right knee pain, difficulty ascending stairs, and knee instability. Given the presentation of symptoms, a physical exam revealed a grade 3 Lachman test, grade 3 anterior drawer test, and grade 2 pivot shift on the affected knee, compared to a grade 1 Lachman and anterior drawer test with no pivot shift on the contralateral side. Additionally, the patient had a history of daily physical therapy with no relief before the consultation, prompting a recommendation for surgery. The patient had no prior history of injury to the affected knee before this incident. After magnetic resonance imaging (Figure 1), the patient proceeded with surgery.

Surgical procedure and postoperative course of treatment

The surgical procedure involved an arthroscopic, all-inside right ACL reconstruction using a quadriceps tendon autograft, partial lateral meniscectomy, and medial meniscus repair. Physical therapy began within three days postoperatively, and the patient was provided with an X-Act range-of-motion brace. She was instructed to bear weight as tolerated, with motion restrictions while using the brace. For the first two weeks, the brace was locked in extension and then gradually adjusted to a range of 0 to 90 degrees until four weeks postoperatively.

The role of postoperative bracing after ACL reconstruction remains a topic of debate. While some studies suggest that functional bracing can improve knee kinematics and offer added protection for patients [5,6], other research, including meta-analyses, has found no significant benefit to bracing. However, these studies also indicate that bracing does not lead to postoperative complications, leaving its necessity controversial [7,8].

Two weeks postoperatively, the patient returned for suture removal, and the surgeon noted a delay in range of motion (ROM) progress. At this follow-up visit, passive ROM measured 5 to 85 degrees, with a five-degree extension block. She was instructed to continue physical therapy and adhere to knee brace restrictions. By four weeks postoperatively, an eight- to nine-degree flexion restriction was noted, raising concerns about worsening ROM. As a result, an extension device was prescribed.

The orthopedic team monitored the patient monthly for the next seven months. During this period, disuse atrophy of the vastus medialis oblique (VMO) and other quadriceps muscles became evident. By the four-month follow-up, the flexion restriction had progressed to 12 degrees, prompting the physician to recommend continued aggressive physical therapy and the ongoing use of the extension device. At the five-month visit, improvement was noted, and the patient transitioned to a hinged knee brace for added stability.

Despite some progress, she had not improved sufficiently to return to the track. Seeking further evaluation, she obtained a second opinion from another orthopedic surgeon within the practice. At this visit, passive ROM had improved from 0 to 130 degrees, while active ROM measured 3 to 95 degrees. However, significant quadriceps atrophy remained, with a 4-cm deficit in the VMO and quadriceps. At the six-month postoperative visit, the patient explored additional nonoperative options for the flexion restriction. A corticosteroid injection was administered in an attempt to release fibrotic tissue, despite the known risks of joint toxicity with repeated use [9].

Unfortunately, one month after the injection, the patient experienced no improvement in knee extension despite continued physical therapy and use of the extension machine. Oral steroid therapy was also attempted but failed to provide relief. Given the lack of progress, the surgeon recommended an arthroscopic partial synovectomy and manipulation under anesthesia (MUA) 10 months post-ACL reconstruction.

**Figure 2:**
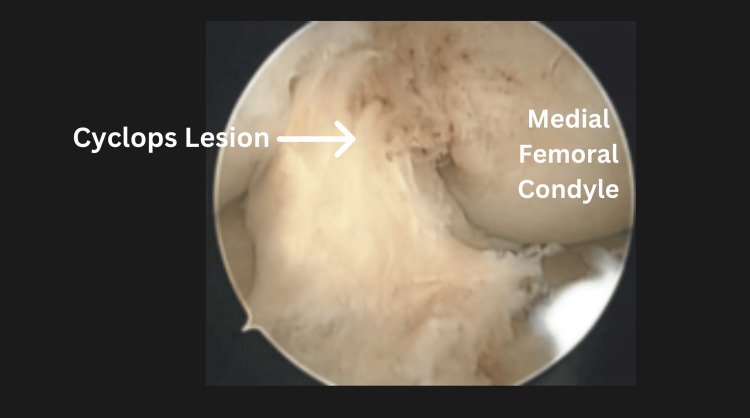
The cyclops lesion

**Figure 3:**
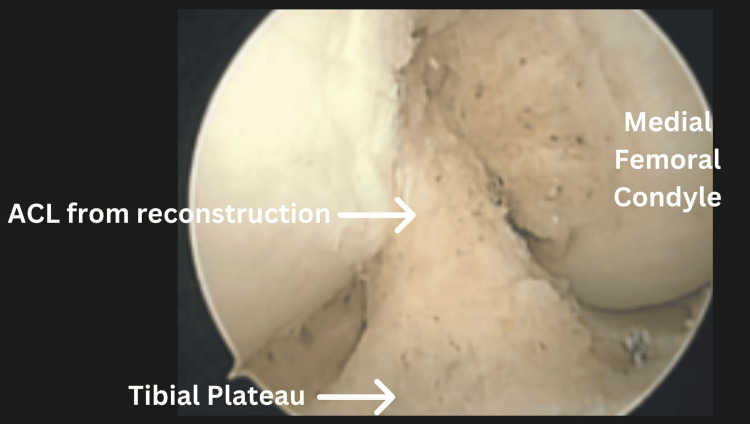
This figure indicates the removal of the cyclops lesion by showing a clear ACL reconstruction, which had been obstructing patellar movement and reducing the range of motion ACL: Anterior cruciate ligament.

During surgery, large cyclops lesions - complications associated with ACL reconstruction - were identified between the ACL graft and the infrapatellar fat pad (Figures 2, 3). Adhesions were also noted in the suprapatellar pouch and gutters. The knee was carefully manipulated, achieving -5 degrees of hyperextension to match the contralateral knee and 140 degrees of flexion. Sixteen days postoperatively, the patient demonstrated significant improvement, achieving a ROM of -3 degrees of hyperextension to 140 degrees of flexion.

Outcome

One month following the arthroscopic partial synovectomy and MUA, the patient was released from the practice and able to return to running track. At this point, she was fully asymptomatic and continued track throughout the rest of her high school career.

## Discussion

In this case, we explored nonoperative treatment options to address a flexion restriction complication following ACL reconstruction in a 16-year-old female track athlete, aiming to avoid a second surgery [10]. Initially, she experienced a minor flexion restriction of less than 10 degrees. Based on available data, she was expected to recover without complications if compliant with the physical therapy protocol. However, despite adhering to an aggressive rehabilitation regimen and using an extension machine, the flexion restriction gradually progressed to 12 degrees.

Although ACL injuries are increasingly common in orthopedic patients, advances in arthroscopic procedures have significantly reduced complications and enabled many individuals to regain near-normal function [11]. Research supports the efficacy of surgical interventions, such as arthroscopic lysis of adhesions and MUA, in restoring ROM and improving patient outcomes when conservative treatments fail [11,12]. This case underscores the importance of early recognition and prompt intervention for postoperative flexion restriction to prevent further deterioration and loss of function. Surgical procedures like lysis of adhesions and MUA can effectively restore knee mobility, alleviate symptoms, and ultimately improve a patient’s quality of life and satisfaction [12].

Additionally, this case highlights the importance of individualized treatment plans, close postoperative monitoring, and timely decision-making when nonoperative methods prove insufficient. For athletes, regaining a full ROM and functional stability is crucial not only for returning to their sport but also for ensuring long-term knee health and overall quality of life. By integrating evidence-based approaches with patient-centered care, orthopedic teams can better manage complex recovery pathways and improve outcomes for individuals facing similar challenges.

## Conclusions

ACL reconstruction remains the gold standard for treating ACL tears in young, active individuals, particularly with advancements in arthroscopic techniques and graft augmentation that minimize complications and help patients regain near-normal function. However, postoperative complications, such as flexion restriction caused by cyclops lesions, can significantly impact recovery and require careful management. While early rehabilitation and adherence to physical therapy protocols are crucial for preventing postoperative stiffness, some cases - like this one - demonstrate that nonoperative measures may not always be sufficient. Despite extensive conservative treatment, including bracing, the use of an extension machine, and corticosteroid therapy, this patient experienced progressive flexion restriction, ultimately necessitating surgical intervention.

The positive outcome of this case highlights the importance of timely recognition and intervention for post-ACL reconstruction complications. Arthroscopic lysis of adhesions and MUA proved effective in restoring full knee mobility, allowing the patient to return to competitive athletics without further limitations. This outcome underscores the necessity of individualized treatment plans, close postoperative monitoring, and prompt surgical intervention when conservative approaches fail. For young athletes, achieving a full ROM and functional stability is critical not only for returning to sports but also for ensuring long-term joint health and overall quality of life. Moving forward, continued research on optimizing postoperative rehabilitation and early detection of complications can further improve outcomes for patients undergoing ACL reconstruction.
